# Nicorandil protects podocytes via modulation of antioxidative capacity in acute puromycin aminonucleoside-induced nephrosis in rats

**DOI:** 10.1152/ajprenal.00144.2022

**Published:** 2022-12-01

**Authors:** Masaki Yamanaka, Yoshifuru Tamura, Emiko Kuribayashi-Okuma, Shunya Uchida, Shigeru Shibata

**Affiliations:** Division of Nephrology, Department of Internal Medicine, Teikyo University School of Medicine, Tokyo, Japan

**Keywords:** ATP-sensitive potassium channel, mitochondrial calcium uniporter, nicorandil, oxidative stress, puromycin-aminonucleoside nephrosis

## Abstract

Nephrotic syndrome, characterized by proteinuria and hypoalbuminemia, results from the dysregulation of glomerular podocytes and is a significant cause of end-stage kidney disease. Patients with idiopathic nephrotic syndrome are generally treated with immunosuppressive agents; however, these agents produce various adverse effects. Previously, we reported the renoprotective effects of a stimulator of the mitochondrial ATP-dependent K^+^ channel (MitK_ATP_), nicorandil, in a remnant kidney model. Nonetheless, the cellular targets of these effects remain unknown. Here, we examined the effect of nicorandil on puromycin aminonucleoside-induced nephrosis (PAN) rats, a well-established model of podocyte injury and human nephrotic syndrome. PAN was induced using a single intraperitoneal injection. Nicorandil was administered orally at 30 mg/kg/day. We found that proteinuria and hypoalbuminemia in PAN rats were significantly ameliorated following nicorandil treatment. Immunostaining and ultrastructural analysis under electron microscopy demonstrated that podocyte injury in PAN rats showed a significant partial attenuation following nicorandil treatment. Nicorandil ameliorated the increase in the oxidative stress markers nitrotyrosine and 8-hydroxy-2-deoxyguanosine in glomeruli. Conversely, nicorandil prevented the decrease in levels of the antioxidant enzyme manganese superoxide dismutase in PAN rats. We found that mitochondrial Ca^2+^ uniporter levels in glomeruli were higher in PAN rats than in control rats, and this increase was significantly attenuated by nicorandil. We conclude that stimulation of MitK_ATP_ by nicorandil reduces proteinuria by attenuating podocyte injury in PAN nephrosis, which restores mitochondrial antioxidative capacity, possibly through mitochondrial Ca^2+^ uniporter modulation. These data indicate that MitK_ATP_ may represent a novel target for podocyte injury and nephrotic syndrome.

**NEW & NOTEWORTHY** Our findings suggest that the mitochondrial Ca^2+^ uniporter may be an upstream regulator of manganese superoxide dismutase and indicate a biochemical basis for the interaction between the ATP-sensitive K^+^ channel and Ca^2+^ signaling. We believe that our study makes a significant contribution to the literature because our results indicate that the ATP-sensitive K^+^ channel may be a potential therapeutic target for podocyte injury and nephrotic syndrome.

## Graphical abstract

**Figure:**
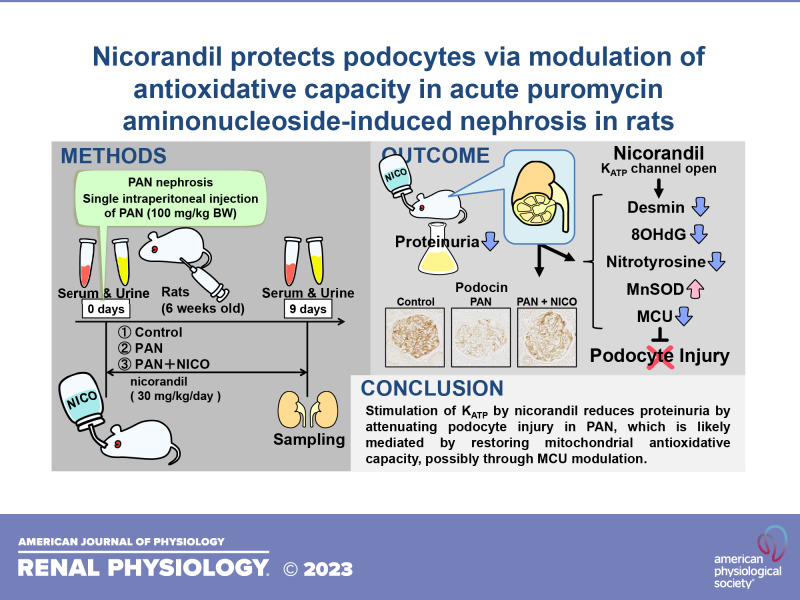

## INTRODUCTION

Nephrotic syndrome, characterized by heavy proteinuria (urinary protein excretion) associated with hypoalbuminemia, edema, and hyperlipidemia, results from impairment of the glomerular filtration barrier of the kidney, with an incidence of 3 new cases per 100,000 each year (1). Patients with idiopathic nephrotic syndrome are generally treated with immunosuppressive agents, including corticosteroids; however, their use is associated with various serious adverse effects such as infection, impaired glucose tolerance, hypertension, osteonecrosis, and mental disorders (2, 3). In addition, not all patients respond to immunosuppressive agents, and steroid-resistant nephrotic syndrome is one of the major causes of end-stage kidney disease that requires renal replacement therapy throughout life (4).

Glomerular epithelial cells, also known as podocytes, wrap around the capillaries of the glomerulus. Podocytes form interdigitating foot processes that are bridged by a specialized adherent junction, known as the slit diaphragm, which serves as the final filtration barrier to prevent the leakage of plasma proteins into the urine (5). Damage to these cells and their detachment from the glomerular base membrane compromise filtration barrier function, leading to proteinuria and progressive kidney failure (6–8). Although the pathogenesis of podocyte injury (podocytopathy) in idiopathic nephrotic syndrome is not entirely clear, several lines of evidence imply the abnormality in Ca^2+^ signaling in podocytes, which has been pursued as a novel therapeutic target for nephrotic syndrome (9, 10). Nonetheless, there are currently no established treatments for podocytopathy besides the use of immunosuppressive agents.

ATP-dependent K^+^ (K_ATP_) channels are present in many tissues, including the cardiovascular system, pancreatic β cells, and the kidney, and regulate diverse physiological processes in the body. K_ATP_ channels are present in cell surface membranes (surface K_ATP_ channels) and mitochondria (MitK_ATP_ channels) (11), and it has been suggested that stimulation of the latter plays a protective role in cardiomyocytes by attenuating mitochondrial Ca^2+^ overload (12–16), a condition that disturbs membrane potential and compromises redox regulation in mitochondria (17, 18).

Nicorandil {2-[(pyridin-3-ylcarbonyl)amino]ethyl nitrate} is a clinically proven antianginal agent that acts as a K_ATP_ channel opener predominantly in the mitochondria (19, 20). Besides the antianginal effects on the cardiovascular system, previous studies have suggested that nicorandil can prevent kidney injury under both acute and chronic conditions (21–24). In particular, we have demonstrated that nicorandil attenuates glomerulosclerosis and tubulointerstitial injury in remnant kidney rats, a chronic kidney disease model (25). Moreover, the protective effects of nicorandil in this model were observed even when an inhibitor of the renin-angiotensin system was coadministered (26). Despite this evidence, the precise mechanisms by which nicorandil confers renoprotection remain unclear. Therefore, we hypothesized that nicorandil prevents podocyte injury by maintaining optimum mitochondrial antioxidant levels. To test this hypothesis, we examined the molecular mechanism of the drug in puromycin aminonucleoside-induced nephrosis (PAN) rats, a model of minimal change nephrotic syndrome resulting from podocyte injury.

## MATERIALS AND METHODS

### Experimental Design

All animal experiments were performed in accordance with the Institutional Animal Care and Use Committee (Animal Ethics Committee, No. 12–053, 18-013) of the Teikyo University School of Medicine. Male Sprague-Dawley rats, weighing 150–200 g, were randomly assigned to the following three groups: *1*) control (*n* = 6 animals), *2*) PAN (*n* = 6 animals), and *3*) PAN with nicorandil treatment (PAN + NICO; *n* = 5 animals). The PAN group received a single intraperitoneal injection of puromycin aminonucleoside (100 mg/kg body wt, Sigma-Aldrich, St. Louis, MO). Animals had free access to food and water. To ensure that equal concentrations of nicorandil were administered, daily water intake volumes were measured, and the concentration of nicorandil was adjusted so that the intake of nicorandil was 30 mg/kg/day. On *days 0*, *4*, and *9*, rats were placed in metabolic cages, and 24-h urine samples were collected to measure urinary protein and creatinine concentrations; in addition, blood samples were also collected on the designated days. All rats were euthanized on *day 9*. The kidneys were excised, and the renal cortex was dissected. Glomeruli were isolated, frozen in liquid nitrogen, and stored at −80°C until use.

### Isolation of the Glomeruli

The glomerular fraction was isolated by sieving as previously described (27, 28). All steps were performed in ice-cold PBS. The cortex of each kidney was separated by macroscopic dissection with a razor blade and carefully minced on a precooled glass dish in sterile PBS. The homogenized tissue was then pushed through a stainless steel sieve, with a pore size of 90 μm, by applying gentle pressure with a stencil. The sieve was rinsed several times with PBS. The tissue below the sieve containing an enrichment of glomeruli was collected and transferred to a sieve with a pore opening of 53 μm. After several washes with 50-mL PBS, the material that remained on top of the sieve (containing the glomeruli) was collected in 50-mL PBS and centrifuged for 8 min at 280 *g*. The supernatant was decanted, and the pellet enriched in glomeruli was resuspended in PBS. The washing step was repeated two to three times until the supernatant became clear.

### Laboratory Experiments

The urine protein content was measured using a protein assay reagent (Thermo Fisher Scientific, Waltham, MA). Serum and urine creatinine, total serum cholesterol, and serum albumin concentrations were enzymatically determined (Oriental Yeast, Tokyo, Japan).

### Periodic Acid-Schiff Staining

Methyl Carnoy’s solution-fixed, paraffin-embedded sections (1-μm thick) were stained with periodic acid-Schiff reagent for light microscopy analysis. Kidneys from all rats were examined histologically. To assess glomerular and tubulointerstitial injury, coronal sections of the kidneys were scanned using NanoZoomer (Hamamatsu Photonics, Shizuoka, Japan). Tubulointerstitial injury was scored semiquantitatively as previously described (29). Ten fields (each field/0.4 mm^2^) of the tubulointerstitium per kidney were evaluated in periodic acid-Schiff-stained biopsy samples. Tubulointerstitial injury was defined as tubular dilation, atrophy, cast formation, sloughing of tubular epithelial cells, and thickening of the tubular basement membrane and was scored on a scale of 0–4 as follows: 0, no tubulointerstitial injury; 1, <25%; 2, 25–50%; 3, 51–75%; and 4, >75% of the tubulointerstitial injury over the total area.

### Immunohistochemistry

Methyl Carnoy’s solution-fixed, paraffin-embedded sections were used for immunohistochemistry, as previously described (26). After deparaffinization, sections were incubated with primary antibodies for 1 h at 37°C. Sections were treated with 3% H_2_O_2_ for 10 min to inactivate endogenous peroxidase activity, followed by treatment with secondary antibodies for 1 h. The signal was visualized by diaminobenzidine. The entire area of the kidney cortex, containing at least 100 glomeruli, was examined. To assess podocin-, desmin-, CD68-, and 8-hydroxy-2-deoxyguanosine (8-OHdG)-positive areas, digital images were analyzed using an Aperio ImageScope (Leica Biosystems, Wetzlar, Germany). In addition, we counted the number of positive cells per glomeruli for Wilms’ tumor-1 (WT-1) protein. The positive area (in %) was determined as diaminobenzidine-positive pixels per total pixels in the glomerular tuft area in each section and was averaged for all glomeruli. Likewise, positive areas for CD68 and 8-OHdG in the interstitium were also determined as the positive area (in %) with five fields per kidney (each field/0.8 mm^2^).

For immunofluorescence, frozen tissues were cut into thin 1.5-μm slices, blocked with 5% animal serum complex, and then incubated at 4°C overnight with rabbit anti-podocin antibody (1:2,000 dilution, ab50339, Abcam, Cambridge, MA) as the primary antibody. After incubation with the secondary antibody for 1 h at 25°C, sections were mounted with Vectashield antifade mounting medium (Vector Labs, Burlingame, CA).

### Western Blot Analysis

Isolated glomeruli were homogenized in cell lysis buffer (Cell Signaling Technology, Danvers, MA) at 4°C. Samples were then processed for SDS-PAGE and electrotransferred onto a polyvinylidene fluoride membrane. After incubation with primary antibody for 1 h at 25°C, the membrane was incubated with secondary antibody conjugated with horseradish peroxidase for 1 h at 25°C. Signals were detected using SuperSignal West Pico Substrate (Thermo Fisher Scientific). The density of each band was determined using Multi Gauge software (Fujifilm, Tokyo, Japan) and expressed as a value relative to the density of the corresponding β-actin (1:5,000 dilution, ab6276, Abcam) band.

### Primary Antibodies for Immunohistochemistry and Western Blot Analysis

We used the following primary antibodies, which have been previously characterized (26): rabbit anti-podocin antibody (1:2,000 dilution) (30), rabbit anti-WT-1 antibody (1:1,000 dilution, sc-192, Santa Cruz Biotechnology, Santa Cruz, CA) (31), mouse anti-desmin antibody (ready-to-use antibody, IS606, Dako, Glostrup, Denmark) (32), mouse anti-rat CD68 antibody (1:100 dilution, MCA341GA, Bio-Rad, Hercules, CA) (33), mouse anti-nitrotyrosine antibody (1:50 dilution for immunohistochemistry and 1:1,000 dilution for Western blot analysis, MAB5404, Millipore, Billerica, MA) (34), rabbit anti-manganese superoxide dismutase (MnSOD) antibody (1:100 dilution for immunohistochemistry and 1:1,000 dilution for Western blot analysis, ADI-SOD-111-D, Enzo Life Sciences, Farmingdale, NY) (35), and mouse anti-8-OHdG antibody (1:40 dilution, MOG-020P, JaICA, Shizuoka, Japan) (36, 37). Mouse anti-sulfonylurea receptor (SUR)2A antibody was obtained from Abcam (1:1,000 dilution, ab174629) (38). The immunogen peptide of the anti-SUR2A antibody and the corresponding amino acid sequence of SUR2B share <40% sequence homology and this antibody does not cross-react with SUR2B. Mouse anti-SUR1/SUR2B antibody was obtained from Thermo Fisher Scientific (1:1,000 dilution, MA5-27636). This antibody recognizes amino acids 1503–1545 at the COOH-terminus of rat SUR2B and does not cross-react with SUR2A (sequence homology < 40%). We also used rabbit antibodies against the mitochondrial Ca^2+^ uniporter (MCU) (1:1,000 dilution, No. 14997, Cell Signaling Technology, Danvers, MA) (39, 40). The signal specificity of this antibody has been previously characterized (41).

### Transmission Electron Microscopy

Transmission electron microscopy was performed according to previously described standard procedures (42). Tissue blocks were fixed in 2.5% glutaraldehyde and embedded in Epon. These samples were fixed using 2% osmium tetroxide, followed by dehydration with increasing concentrations of ethanol. Samples were sectioned 0.6 μm apart. Samples from each glomerulus were examined under a H7520 electron microscope (Hitachi, Tokyo, Japan). Analysis of podocyte foot processes was performed at a magnification of ×30,000. The number of filtration slits per 10 μm length of glomerular basement membrane was calculated using transmission electron microscopy.

### Cell Culture

Immortalized mouse podocytes (5) were cultured in RPMI-1640 medium (Gibco-Invitrogen, Carlsbad, CA) supplemented with 10% FBS, penicillin (100 U/mL) and streptomycin (100 µg/mL). After induction of differentiation at 37°C for 2 wk, SUR2B and MCU expression in the mitochondrial fraction was examined. The protocol was adapted from Itahana et al. (43). Briefly, podocytes were collected by centrifugation at 800 *g* for 5 min and washed twice with cold PBS. Cells were then lysed in cold hypotonic buffer and incubated on ice for 3 min. Cellular debris containing nuclei and mitochondria was removed by centrifugation (13,000 *g*) for 10 min at 4°C. The supernatants were transferred to a fresh tube (cytoplasmic fraction). The cell pellets were further washed twice with cold hypotonic buffer and then pelleted by centrifugation (13,000 *g*, 2 min). The pellets (containing mitochondria) were lysed with cold 0.5% Triton X-100/hypotonic buffer and incubated on ice for 1 min. Subsequently, cellular debris was removed (13,000 *g*, 5 min, 4°C), and the supernatants were transferred to a fresh tube (mitochondrial fraction). Total cell lysate and cytoplasmic and mitochondrial fractions were analyzed by Western blot analysis with antibodies against SUR2B and MCU.

### Statistical Analysis

All values are expressed as means ± SD. Group differences were analyzed by Welch's ANOVA test followed by Dunnett’s T3 post hoc test for multiple comparisons or by one-way ANOVA and post hoc Tukey’s test. Time-dependent data were analyzed using repeated-measures ANOVA followed by the Bonferroni multiple comparison test. All statistical analyses were performed using GraphPad Prism 9 (GraphPad Software, San Diego, CA). Statistical significance was set at *P* < 0.05.

## RESULTS

### Nicorandil Attenuates Proteinuria and Hypoalbuminemia in PAN Rats

**Figure 1.**
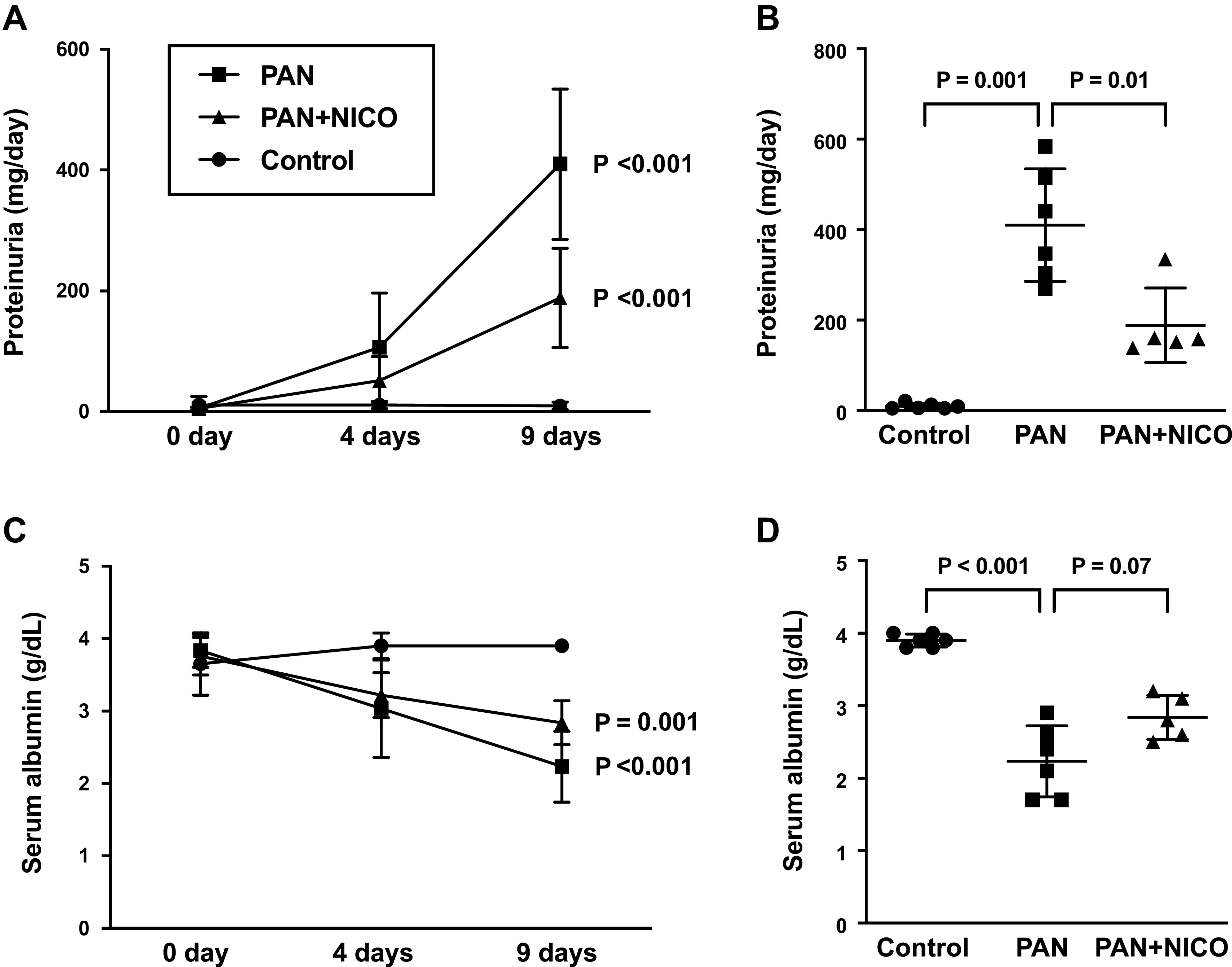
Antiproteinuric effect of nicorandil in puromycin aminonucleoside-induced nephrosis (PAN) rats. *A* and *B*: urinary protein excretion was evaluated using 24-h urine collection samples. Data are expressed as means ± SD. *A*: proteinuria during the 9-day experimental period. *P* values vs. *day 0*. *B*: proteinuria at *day 9*. *C*: serum albumin level during the 9-day experimental period. *P* values vs. *day 0*. *D*: serum albumin level at *day 9*. PAN + NICO rats, PAN rats treated with nicorandil.

We observed massive proteinuria in PAN rats compared with control rats on *days 4* and *9*. However, nicorandil administration significantly reduced urinary protein excretion on *day 9* (Fig. 1, *A* and *B*). In addition, nicorandil had a tendency to prevent hypoalbuminemia on *day 9* (Fig. 1, *C* and *D*). Analogous to human nephrotic syndrome, PAN rats showed a marked increase in total cholesterol levels (Supplemental Table S1). Although serum total cholesterol levels were low in PAN + NICO rats compared with PAN rats, the difference was not statistically significant due to high variability.

### Effect of Nicorandil on Renal Histology

**Figure 2.**
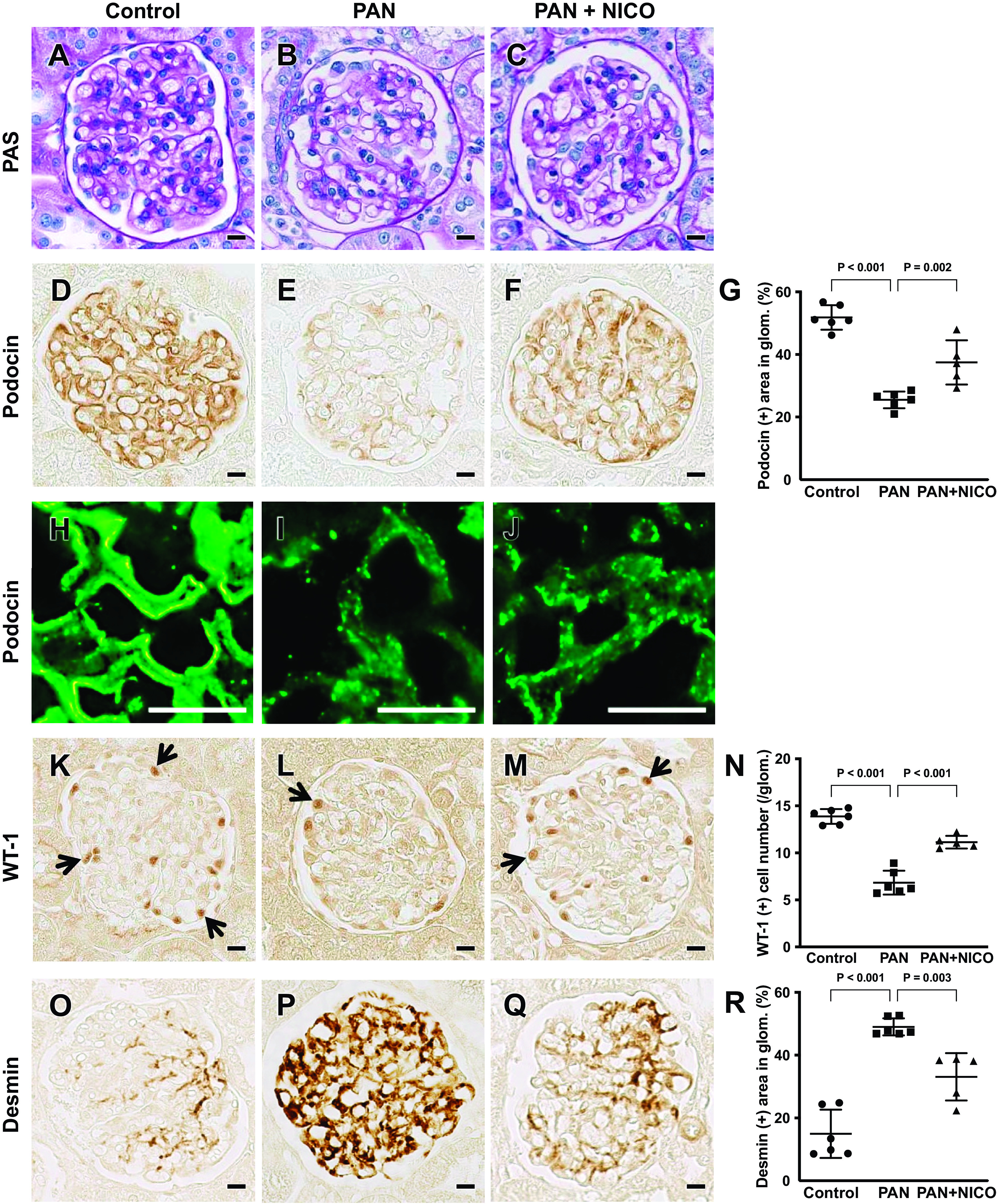
Nicorandil ameliorates podocyte damage in puromycin aminonucleoside-induced nephrosis (PAN) rats. Periodic acid-Schiff (PAS) staining showed no histopathological changes in glomeruli in the PAN group (*B*) compared with the control (*A*) and PAN + nicorandil (PAN + NICO; *C*) groups. *D–N*: podocin (*D–F* and *H–J*; localization of podocin in glomeruli elucidated using immunofluorescence) and Wilms’ tumor-1 (WT-1; *K–M*) levels were reduced in the PAN group (*E*, *I*, and *L*) compared with the control group (*D*, *H*, and *K*); however, these changes were attenuated in the PAN + NICO group (*F*, *J*, and *M*). *G* and *N*: quantification of podocin (*G*) and WT-1 (*N*). *O–Q*: immunostaining for desmin, a marker for podocyte injury. *R*: quantification of desmin staining in glomeruli. Data are expressed as means ± SD. Scale bars = 10 μm.

**Figure 3.**
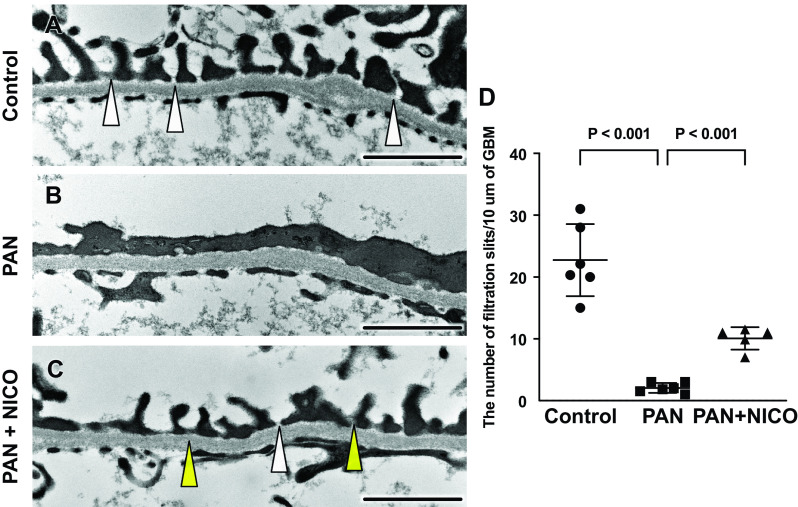
Assessment of filtration slit widths of the glomerular basement membrane by transmission electron microscopy. *A–C*: transmission electron micrographs of podocyte foot processes in the glomeruli of indicated animals. Foot process effacement (arrowheads) was more pronounced in puromycin aminonucleoside-induced nephrosis (PAN) rats (*B*) than in control rats (*A*). In PAN + nicorandil (PAN + NICO) rats (*C*), the structure of the foot processes was relatively preserved (white arrowhead), although some effaced foot processes were observed (yellow arrowhead). *D*: number of filtration slits of the glomerular basement membrane. Data are expressed as means ± SD. Scale bars = 1 μm.

Previous studies have shown that the PAN model does not show apparent changes in glomerular structure under light microscopy; however, podocyte injury has been demonstrated to occur by immunohistochemical and ultrastructural analyses (44). As expected, no obvious difference was noted in glomeruli in periodic acid-Schiff-stained kidney sections among the three groups (Fig. 2, *A–C*). However, immunostaining for the podocyte markers podocin and WT-1 revealed that the levels of both parameters were reduced in PAN rats compared with control rats (Fig. 2, *D*, *E*, *H*, *I*, *K*, and *L*). Conversely, podocytes in PAN rats showed an increase in desmin expression (Fig. 2, *O* and *P*), a marker of podocyte injury (42). Administration of nicorandil significantly alleviated these changes (Fig. 2, *F*, *J*, *M*, and *Q*). Similarly, levels of both podocin and WT-1 were significantly reduced (both *P* < 0.001) in PAN rats compared with control rats; they were also significantly reduced (podocin: *P* = 0.002 and WT-1: *P* < 0.001) in PAN + NICO rats (Fig. 2, *G* and *N*). Levels of desmin were significantly increased (*P* < 0.001) in PAN rats compared with control rats, which was significantly alleviated (*P* = 0.003) in PAN + NICO rats (Fig. 2*R*). To directly demonstrate the protective effects of nicorandil on glomerular podocytes, the podocyte ultrastructure was examined using transmission electron microscopy. Figure 3*A* shows the normal podocyte architecture in control rat kidneys. In PAN rats, the foot processes were disturbed and showed marked effacement (Fig. 3*B*). However, in PAN + NICO rats, the structure of foot processes was relatively maintained compared with PAN rats (Fig. 3*C*), which was confirmed by semiquantitative analysis of the filtration slit widths (Fig. 3*D*). These data indicate that nicorandil protects against podocyte injury in PAN rats, resulting in reduced proteinuria and increased serum protein levels.

**Figure 4.**
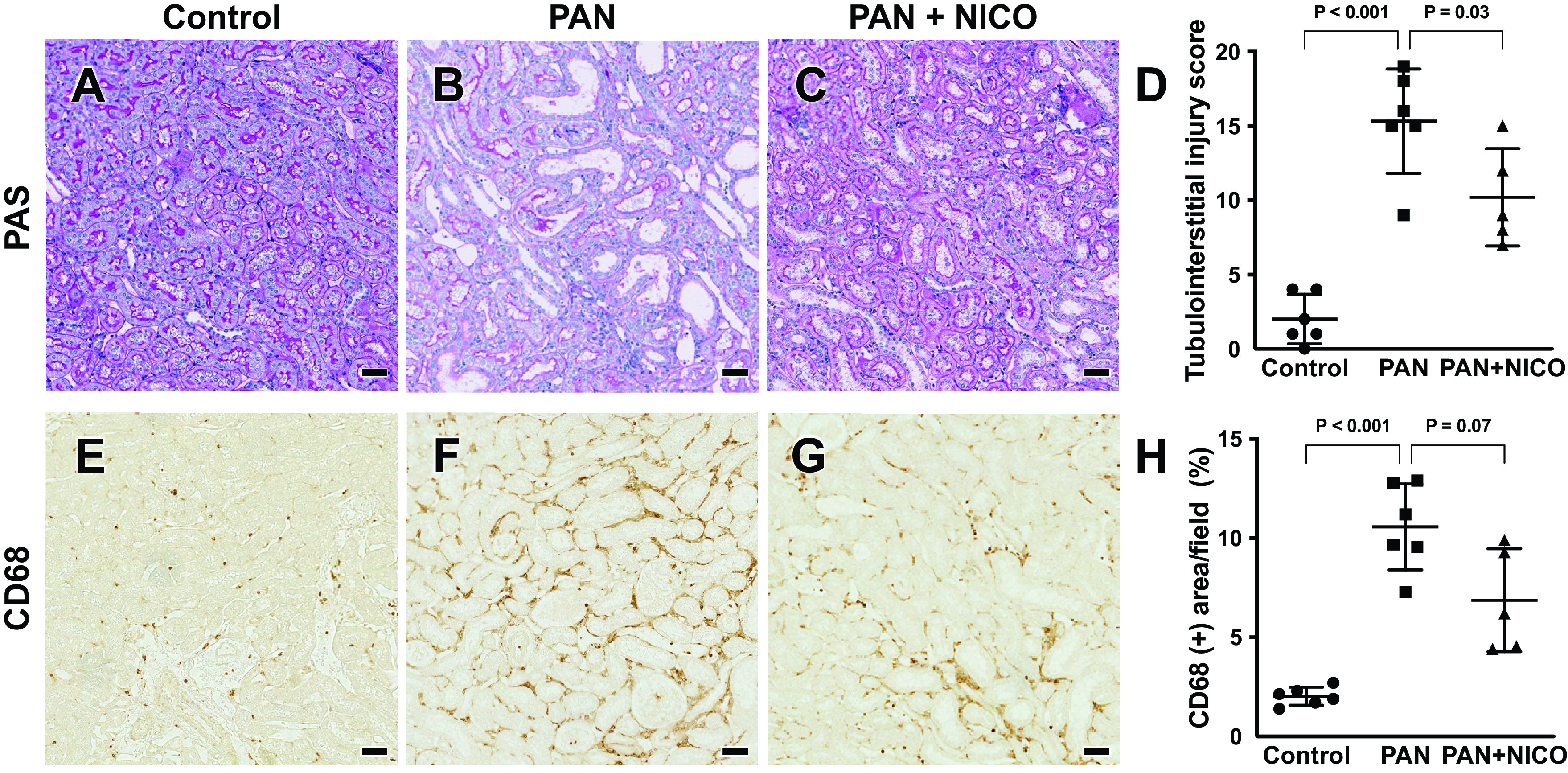
Attenuation of tubulointerstitial injury by nicorandil in puromycin aminonucleoside-induced nephrosis (PAN) rats. Periodic acid-Schiff (PAS) staining showed dilated renal tubules in the PAN group (*B*) compared with the control (*A*) and PAN + nicorandil (PAN + NICO; *C*) groups. *D*: quantification of tubulointerstitial injury (see materials and methods for details). *E–G*: infiltration of CD68 cells (markers of mononuclear cells) was pronounced in the PAN group (*F*) compared with the control group (*E*). *G*: infiltration of CD68-positive cells was attenuated in the PAN + NICO group. *H*: quantification of CD68-positive areas. Data are expressed as means ± SD. Scale bars = 50 μm.

The PAN model exhibits tubulointerstitial injury secondary to heavy proteinuria and protein loading in renal tubules (45). Therefore, we evaluated whether the reduction in proteinuria resulted in the attenuation of tubular damage and tubulointerstitial inflammation. As shown in Fig. 4, *A* and *B*, PAN rats exhibited tubulointerstitial injury characterized by tubular dilation, protein casts, and macrophage infiltration. CD68 staining also demonstrated an increased number of inflammatory cells in the kidneys in this model (Fig. 4, *E* and *F*). PAN + NICO rats showed amelioration of these tubulointerstitial alterations (Fig. 4, *C*, *D*, *G*, and H), further supporting the renoprotective effect of nicorandil in this model.

### Nicorandil Attenuates Oxidative Stress in PAN Rats

**Figure 5.**
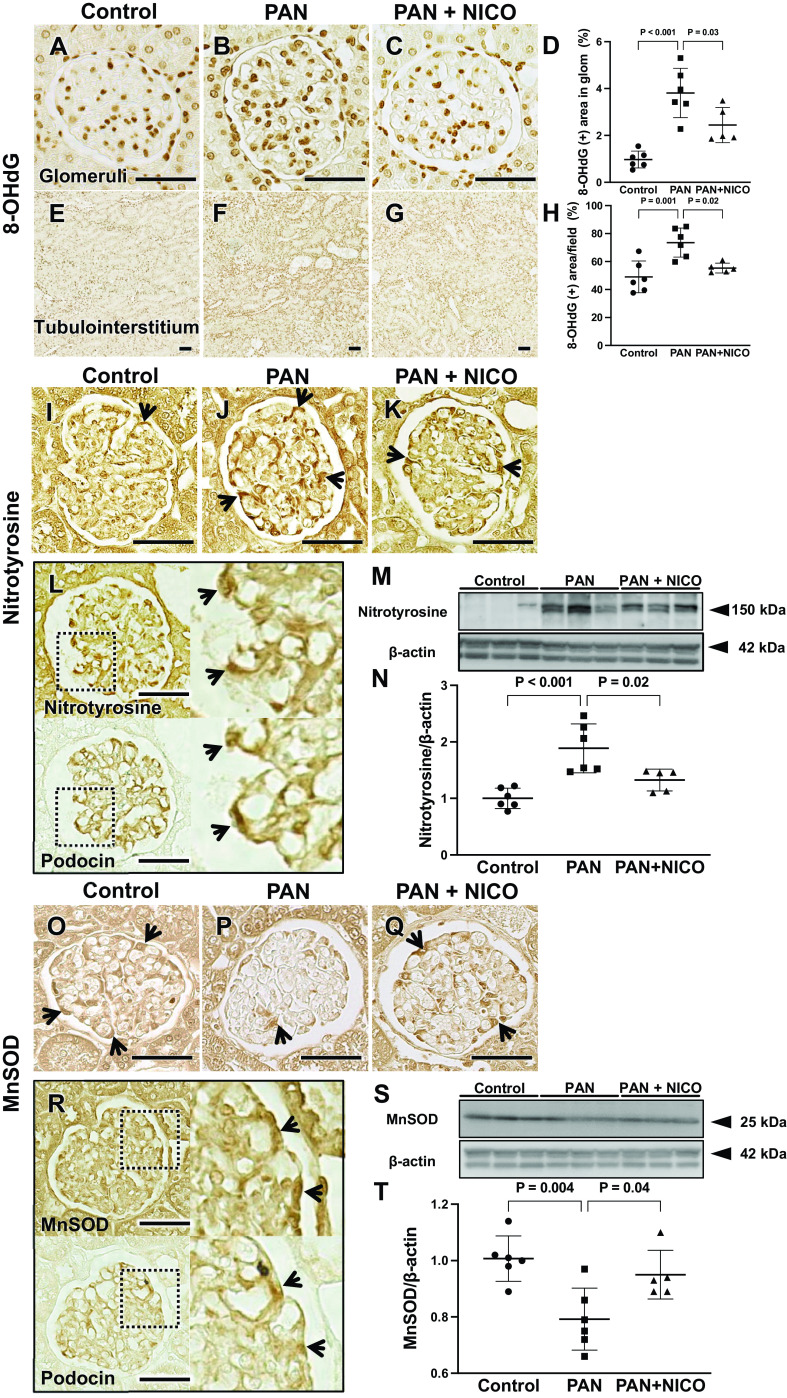
Nicorandil reduces oxidative stress in puromycin aminonucleoside-induced nephrosis (PAN) rats. Glomerular 8-hydroxy-2-deoxyguanosine (8-OHdG) deposition (brown) was more prominent in the PAN group (*B*) than in the control (*A*) and PAN + nicorandil (PAN + NICO; *C*) groups. *D*: quantification of the intensity of the immunohistochemical signal for 8-OHdG. Tubulointerstitial 8-OHdG deposition (brown) was more prominent in the PAN group (*F*) than in the control (*E*) and PAN + NICO (*G*) groups. *H*: quantification of the intensity of the immunohistochemical signal for 8-OHdG. Immunohistochemistry demonstrated that nitrotyrosine (arrow) was expressed in the glomerulus. The number of positive cells was higher in the PAN group (*J*) than in the control (*I*) and PAN + NICO (*K*) groups. *L* and *R*: immunohistochemistry with serial sections. In *L*, nitrotyrosine (arrow) overlapped with podocin (arrow), respectively. *M*: Western blot analysis of glomerular protein lysates demonstrated that nitrotyrosine levels were significantly higher in the PAN group than in the control group; however, nitrotyrosine levels were significantly attenuated in the PAN + NICO group. *N*: quantification of nitrotyrosine levels. Immunohistochemistry demonstrated that manganese superoxide dismutase (MnSOD; arrow) was expressed in the glomerulus. The number of positive cells was lower in the PAN group (*P*) than in the control (*O*) and PAN + NICO (*Q*) groups. In *R*, MnSOD (arrow) overlapped with podocin (arrow), respectively. *S*: Western blot analysis of MnSOD in glomerular protein lysates. MnSOD levels were significantly lower in the PAN group than in the control group. The reduction was attenuated in the PAN + NICO group. *T*: quantification of MnSOD levels. Data are expressed as means ± SD. Scale bars = 50 μm.

To investigate the mechanisms underlying the protective effects of nicorandil on glomerular podocytes, we examined oxidative stress, a well-known mediator of kidney disease (46). Immunostaining of the oxidative stress marker 8-OHdG demonstrated that 8-OHdG levels of glomeruli and the tubulointerstitium were higher in the kidneys of PAN rats than in control rats, and this increase was attenuated by nicorandil (Fig. 5, *A–H*). Similarly, levels of nitrotyrosine, another marker of oxidative stress, were significantly higher in glomeruli of rats in the PAN group than in the glomeruli of rats in control group. However, the increase in nitrotyrosine levels was significantly attenuated by nicorandil (Fig. 5, *I–K, M*, and *N*). Nitrotyrosine overlapped with podocin in serial sections (Fig. 5*L*). To further clarify the mechanisms by which nicorandil blocks oxidative stress, levels of the antioxidant enzyme MnSOD were examined. We found that MnSOD abundance was lower in glomeruli of PAN rats than in control rats and was restored following nicorandil treatment (Fig. 5, *O–Q*, *S*, and *T*). MnSOD overlapped with podocin in serial sections (Fig. 5*R*).

### Possible Involvement of SUR2B and MCU

**Figure 6.**
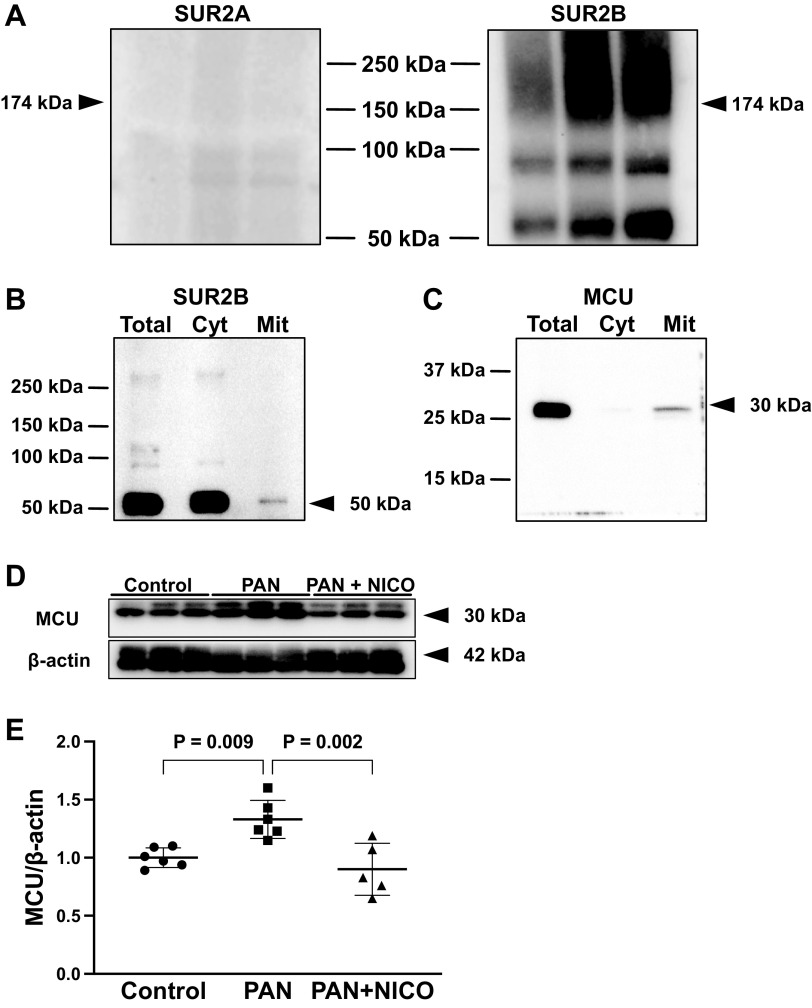
Expression of sulfonylurea receptor (SUR)2 and mitochondrial Ca^2+^ uniporter (MCU) in rat glomeruli and human podocytes. *A*: Western blot analysis of glomerular protein lysates using SUR2A and SUR2B antibodies. *B*: Western blot analysis of podocyte protein lysates using SUR2B antibodies. *C*: Western blot analysis of podocyte protein lysates using MCU antibodies. *D*: Western blot analysis of glomerular protein lysates demonstrated that MCU levels were significantly higher in the puromycin aminonucleoside-induced nephrosis (PAN) group than in the control group. Nicorandil treatment significantly attenuated MCU levels. *E*: quantification of MCU abundance. Cyt, cytoplasmic fraction; Mit, mitochondrial fraction; PAN + NICO rats, PAN rats treated with nicorandil; Total, total cell lysate. Data are expressed as means ± SD.

K_ATP_ channels are composed of an inwardly rectifying K^+^ channel subunit and SUR, the latter of which is a binding site for nicorandil. Previously, we have shown that podocytes express SUR2 (25); however, it is unclear whether they express the cardiac type of SUR (SUR2A) or vascular smooth muscle type of SUR (SUR2B). SUR2A and SUR2B result from alternate splicing of the terminal exon, with the 42 amino acids at the COOH-terminus differing between the two isoforms. Therefore, we attempted to delineate the expression of SUR2 in glomeruli using antibodies that recognize the COOH-terminal portion of these proteins. As shown in Fig. 6*A*, we detected several bands using Western blot analysis for SUR2B, likely representing the core- and complex-glycosylated forms (47). However, these signals were not observed with Western blot analysis using the SUR2A antibody, suggesting that the effects of nicorandil are likely mediated by SUR2B. In addition, SUR2B was present in the mitochondrial fraction of podocytes (Fig. 6*B*).

We then evaluated the mechanism by which nicorandil increases MnSOD levels. Given that both MitK_ATP_ and MnSOD are present in the mitochondria, we reasoned that these effects are mediated by mitochondrial proteins. Moreover, a previous study has indicated that the protective effects of nicorandil are attributable to the dissipation of mitochondrial membrane potential and a reduction in mitochondrial Ca^2+^ overload (16). Therefore, we examined the localization and levels of MCU, a key regulator of Ca^2+^ influx into the mitochondria (48). Our data showed that MCU was present in the mitochondrial fraction of podocytes (Fig. 6*C*). In addition, MCU levels in glomeruli were significantly higher in PAN rats than in control rats. However, this increase was significantly attenuated by nicorandil (Fig. 6, *D* and *E*).

## DISCUSSION

Previous studies have demonstrated that nicorandil reduces urinary albumin excretion in models of chronic kidney disease and diabetic kidney disease (23, 25, 26). In this study, we used PAN rats to examine whether the protective effects of nicorandil are attributable to the protection of glomerular podocytes. We demonstrated that nicorandil attenuated proteinuria and prevented the progression of hypoalbuminemia, which are pivotal features of nephrotic syndrome. We also demonstrated that podocyte injury was attenuated by nicorandil, establishing that podocyte protection accounts for the previously reported renoprotective effects of nicorandil; however, the detailed mechanism remained unclear thus far.

In the present study, we found that the protective effect of nicorandil on podocytes in the PAN model can be ascribed to the restoration of mitochondrial antioxidative capacity. Nicorandil appears to be a fairly selective MitK_ATP_ channel opener (19). In the present study, MnSOD and MCU were suspected to be involved in the effects of nicorandil. Therefore, we suspected that the primary effects are mediated through MitK_ATP_ channels. Consistent with our previous studies (25, 26), the present study demonstrated that the MitK_ATP_ channel opener nicorandil reduces oxidative stress and increases MnSOD levels. In our previous study using cultured podocytes, we found that nicorandil directly induces MnSOD expression (26). Conversely, the K_ATP_ channel blocker glibenclamide decreased MnSOD levels, highlighting the role of the MitK_ATP_ channel in this mechanism (26). These effects are accompanied by changes in sirtuin 3 (SIRT3) levels (26), a mitochondrial deacetylase that regulates MnSOD activity (49). The present study is in line with these observations and indicates that the MitK_ATP_ channel restores antioxidative capacity through MnSOD induction in glomerular cells, including podocytes.

We infer that the MCU may serve as an upstream regulator of MnSOD. Mitochondria not only generate ATP but are also the main source of cellular oxidative stress; they also possess various antioxidative enzymes, such as MnSOD, for redox regulation. Furthermore, mitochondria uptake and store large amounts of Ca^2+^ from the cytosol. Ca^2+^ and ROS overload may result in the formation of the mitochondrial permeability transition pore, which leads to apoptosis or necrosis of the cell (50). MCU, which is localized in the inner mitochondrial membrane, is the main determinant of Ca^2+^ influx that controls mitochondrial function (51). Of note, a recent study has indicated that dysregulation of MCU downregulates the SIRT3-MnSOD pathway through aberrant Ca^2+^ signaling in mitochondria, resulting in increased ROS production (52). Consistent with these observations, our study suggests that upregulation of MCU is accompanied by downregulation of MnSOD in the PAN model. Moreover, given that nicorandil attenuates the upregulation of MCU in PAN rats, these data may also provide a biochemical basis for the interaction between the MitK_ATP_ channel and Ca^2+^ signaling in mitochondria (14–16). Although the pathophysiological role of MCU in kidney diseases remains largely unclear, a previous report has shown that an MCU inhibitor, ruthenium red, decreases proteinuria and attenuates podocyte foot process effacement in adriamycin-induced nephropathy (40). Interestingly, in a recent study, it was observed that patient sera and factors implicated in idiopathic nephrotic syndrome induce marked oxidative stress in podocytes that results in increased expression of ion channels (transient receptor potential cation channel subfamily C member 6) implicated in Ca^2+^ dynamics. The findings of this study might explain the relationship between the results of the present study with human nephrotic syndrome. The role of MCU and Ca^2+^ in podocyte injury requires further investigation (53).

The localization of SUR in the glomerulus is not entirely clear. A previous study has detected the SUR2B transcript, but not the SUR2A transcript, in kidney and mesangial cells (54). It is unclear whether SUR2B is present in glomeruli, although previous studies have shown the presence of SUR2 in podocytes and mesangial cells (23, 25). Our data showed that SUR2B protein was present in the glomeruli in vivo. In vitro, SUR2B protein was present in the mitochondrial fraction of podocytes, which is partly consistent with the results of previous studies (23, 55). These results suggest that nicorandil potentially acts on the mitochondria of podocytes.

The present study has several limitations. First, we did not evaluate the contribution of nitric oxide (NO). NO formation is increased during PAN-induced nephrotic syndrome; however, NO is not involved in the development of glomerular injury (56). Although given that nicorandil not only acts as an opener of the K_ATP_ channel but also as a donor for NO, and given the protective effects of NO in cardiovascular and inflammatory diseases, it is unclear how the modulation of NO contributes to the protective effects of nicorandil in PAN rats. Second, nicorandil is not specific for MitK_ATP_ channels. Besides, K_ATP_ channels are expressed in various other locations in the kidney. However, we evaluated only glomerular proteins and lysates from the podocyte cell line. Furthermore, the present study was not able to provide an electrophysiological assessment of the MitK_ATP_ channel and MCU. The mechanism by which nicorandil inhibits MCU via MitK_ATP_ warrants further investigation. Finally, only a few studies have attempted to assess mitochondrial morphology in PAN using electron microscopy (57–60). In PAN tissues, some of the mitochondrial cristae were absent, the matrix density was low in some areas, and the outer and inner membranes were separated (57). PAN resulted in mitochondrial division (58, 59). However, such features were not apparent in our model, presumably because they were not evaluated sufficiently in the present study.

### Conclusions

Stimulation of K_ATP_ channels by nicorandil reduces proteinuria by attenuating podocyte injury in PAN, possibly by restoring the mitochondrial antioxidative capacity. These data indicate that the K_ATP_ channel may represent a novel target for glomerular podocyte injury and idiopathic nephrotic syndrome.

## DATA AVAILABILITY

Data will be made available upon reasonable request.

## SUPPLEMENTAL DATA

10.6084/m9.figshare.21569784.v1 Supplemental Table S1: https://doi.org/10.6084/m9.figshare.21569784.v1.

## GRANTS

This work was supported in part by Ministry of Education, Culture, Sports, Science and Technology of Japan Grants 19H03678 and by Advanced Comprehensive Research Organization Research Grants from Teikyo University (to S.S.).

## DISCLOSURES

No conflicts of interest, financial or otherwise, are declared by the authors.

## AUTHOR CONTRIBUTIONS

M.Y. and Y.T. conceived and designed research; M.Y. and Y.T. performed experiments; M.Y., Y.T., and E.K-O. analyzed data; M.Y., Y.T., E.K-O., and S.S. interpreted results of experiments; M.Y. and Y.T. prepared figures; M.Y., Y.T., and S.S. drafted manuscript; Y.T., S.U., and S.S. edited and revised manuscript; Y.T., S.U., and S.S. approved final version of manuscript.
